# An oral French maritime pine bark extract improves hair density in menopausal women: A randomized, placebo‐controlled, double blind intervention study

**DOI:** 10.1002/hsr2.1045

**Published:** 2023-01-06

**Authors:** Carr Cai, Bill Zeng, Lydia Lin, Miranda Zheng, Carolina Burki, Susanne Grether‐Beck, Jean Krutmann

**Affiliations:** ^1^ Intertek Testing Services Ltd Shanghai China; ^2^ Horphag Research Geneva Switzerland; ^3^ IUF ‐ Leibniz Research Institute for Environmental Medicine Düsseldorf Germany; ^4^ Medical Faculty Heinrich Heine University Düsseldorf Germany

**Keywords:** female pattern, hair density, microcirculation, placebo‐controlled, Pycnogenol®

## Abstract

**Background and Aims:**

Female pattern hair loss affects females of all ages with a trend to increase after menopause. This disorder may have significant psychological impact and lead to anxiety and depression.

**Objective:**

In a single center, double blind, randomized, placebo‐controlled study, the effects of oral Pycnogenol® intake (3 × 50 mg/day for a total of 6 months) on hair density, scalp microcirculation, and a variety of skin physiological parameters was studied in Han Chinese menopausal women (*N* = 76) in Shanghai, China.

**Methods:**

Measurements were taken at the beginning and after 2 and 6 months, respectively. Hair density was determined by digital photographs and further evaluated by Trichoscan software. Transepidermal water loss was measured by a humidity sensor in a closed chamber on the skin surface. Changes in microcirculation were detected as resting flux on the scalp by reflection photoplethysmography.

**Results:**

Pycnogenol® intake significantly increased hair density by 30% and 23% after 2 and 6 months of treatment, respectively, as detected by Trichoscan® evaluation of digital photographs. Interestingly, photoplethysmography revealed that this beneficial effect was associated with a decrease in resting flux of the scalp skin, which might indicate an improvement of microcirculation. None of these effects were observed in the placebo taking group. In addition, a significant transient decrease of transepidermal water loss was observed in scalp skin under Pycnogenol,® but not placebo treatment.

**Conclusion:**

Oral intake of Pycnogenol® might have the potential to reduce hair loss in postmenopausal women.

## 1 INTRODUCTION

Female pattern hair loss (FPHL) is a general diffuse hair thinning over the central scalp varying among population groups which ordinarily increases with age.^1^ Although no robust epidemiological data are available, due to a lack of unifying criteria, FPHL is very likely to be a frequent disorder. Accordingly, Gan and Sinclair^2^ studied the prevalence of balding among Caucasian women in Maryborough, Victoria, Australia. Based on a five level staging with level one being the normal status these authors found 23% of middle aged women in an age range from 40 to 49 years suffered from mild hair loss, and that this percentage increased in older ages. According to Williams et al.,^3^ thinning hair in women is commonly associated with the menopause. Hormonal changes in menopause affect growth rate, percentage anagen, hair diameter, and diameter distribution, while scalp hair density decreases with age leading to heightened perception of decreased scalp coverage.^4^ In addition, age‐related changes in dermal sheath and dermal fibroblasts might contribute to the observed impact on hair growth.^5^ In the United States, approximately 21 million women are affected.^6^ Recently, the prevalence of FPHL in Asian women was reviewed. Starace et al. found a similar prevalence, again with an age‐related trend, which, however, seems to be lower than in Caucasians.^1^, ^7^, ^8^, ^9^ Although FPHL is no disease, it is regarded as a disorder with a significant psychological impact, which may lead to anxiety and depression.^1^, ^10^, ^11^

Human hair is synthesized in the hair follicle through a growth cycle including a long phase of active fiber production (anagen) and a shorter resting phase (telogen), interspaced by short lasting phases or regression and neomorphogenesis.^12^ On the scalp, hair remains in the anagen phase for a period of 2‐ to 6‐years, whereas that of telogen is approximately 100 days.^13^ Approximately 15%−20% of scalp hairs are usually in the telogen phase.^14^ FPHL is characterized by a follicular regression, which in some cases is associated with elevated androgen levels or microinflammation.^15^ An involvement of vascularization is suggested by observations indicating a decreased subcutaneous blood flow. Also, immunohistopathological studies comparing VEGF localization in alopecia skin versus normal skin indicate decreased angiogenesis.^16^, ^17^ Along the same lines, Minoxidil, a potassium channel opener, which has been FDA approved for the treatment of alopecia, is supposed to enhance angiogenesis around the hair follicle.^18^

Pycnogenol® is a proprietary bark extract from the French maritime pine tree. It mainly contains 70 ± 5% procyanidins built from condensed monomers of catechin and epicatechin. Based on a recent update by the American Botanical Council of the Scientific and Clinical Monograph for Pycnogenol® it is stated that an independent panel of toxicology experts has classified PYC as generally recognized as safe (GRAS) based on clinical safety and preclinical toxicology data.^19^, ^20^ Oral intake of Pycnogenol® is best known for its antioxidative and anti‐inflammatory effects.^16^ In addition, Pycnogenol® intake also improves microcirculation as was shown in patients suffering from venous insufficiency such as diabetic ulcers and diabetic microangiopathy. Accordingly, Pycnogenol® intake decreased the skin flux at rest, and this decrease was associated with an increase in pO_2_ and a decrease in pCO_2_.^17^, ^21^ This effect is not specific for this patient group, because even in healthy young subjects, intake of Pycnogenol® was accompanied by a decrease in the resting flux.^22^ Positive effects of Pycnogenol® intake on endothelial function were also observed in patients with stable coronary artery disease in a double blind, randomized placebo controlled cross over study, showing a significant increase in flow mediated dilation after 8 weeks.^23^

In the present randomized, double‐blind, placebo‐controlled study we asked if oral administration of 3 × 50 mg Pycnogenol® a day over a 6‐month period will affect (i) total hair density and (ii) scalp microcirculation in postmenopausal Chinese women.

## 2 MATERIALS AND METHODS

### 2.1 Study subjects

The study was conducted by the Intertek Testing Services Ltd, during the time period from August 28, 2018 to June 3, 2019. The study was approved by the Ethics Committee of Intertek China, Intertek Testing Services Ltd. Shanghai, China with number 2018 EC 01. The design was a single‐center, double blind, randomized, placebo‐controlled intervention study with no further follow‐up. The purpose of the study was to assess the efficacy of regular oral intake of Pycnogenol® to improve hair density in Chinese menopausal (>1 year since final menstrual period) female volunteers aged 45−60 years. Inclusion criteria were healthy volunteers as assessed by a general physical examination, who showed no sign of skin disease with special reference to hair and scalp. Subjects had no known allergy or intolerance to any component of the test preparations. They had actively cooperated to participate in the study, agreed to follow the instructions of the investigator and to visit the study center at the agreed times; they had also agreed not to use any additional nutritional supplements during the study, to retain the same hair style, hair color, and hair regimen throughout the study. All study subjects signed an informed consent form after the nature and the purpose of the study had been explained to them. Baseline characteristics of participants are given in Table 1

**Table 1:** Baseline characteristics of the participants per (protocol)

	Placebo	Pycnogenol®
Number	30	33
Female	30	33
Mean age in years (SD)	53.7 (3.3)	54.2 (4.0)
Minimal age	45	46
Maximal age	59	60

Exclusion criteria were: age <45, >60 years, known hypersensitivity to any components of the study preparations, any use of hormone replacement therapy, any use of different oral nutritional supplements and/or vitamin supplementation 2 months before and during the study, topical use of any dermatological drug or cosmetic preparation containing ingredients with hair regrowth purposes (e.g., caffeine or dedicated plant extracts) or which were explicitly directed at the treatment of male pattern baldness on the test areas, any systemic or topical medication likely to interfere with the study purposes, particularly antihistamine drugs within a week, immunosuppressive or immunomodulator therapy such as corticosteroids within a month before study enrollment, any use of topical dermatological drug or cosmetic preparation (e.g., sunscreens) on the test area 5 days prior of commencing or during the study, participation in another clinical study/use of experimental drug(s) within the previous 30 days and/or during the study, cigarette consumption higher than four cigarettes per day, history or evidence of drug or alcohol abuse (more than three standard glasses alcoholic beverages per day), and impaired cooperation or unwillingness to satisfactorily participate in the study.

### 2.2 Study materials

The test products were Pycnogenol® and placebo capsules; the latter looked identical to Pycnogenol,® but did not contain any active substances and only consisted of cellulose. Both were provided by Horphag Research. All test products were stored at room temperature in a secure area before study start. All supplements were supplied in identically packed, coded containers. For the intervention, test products were orally administered in a dose of 1 pill three times a day with the meals for a total of 6 months. The Pycnogenol® dose was 150 mg (3 × 50 mg) per day.

### 2.3 Study design

At baseline, hair status and treatment responses were documented by taking digital photographs, which were further evaluated by employing Trichoscan® software (Tricholog GmbH & Datinf GmbH) as described by Hoffmann.^24^ In addition, skin physiological parameters of the scalp such as determination of peripheral microcirculation by photoplethysmography (Doppler flow detector; Smart dop 45; in PPG mode; Hadeco; software Smart‐V‐Link™), transepidermal water loss (TEWL; Vapometer; Delfin), and hydration (Dermalab; Cortex Technology) were performed following the recommendations.^25^ With the PG21 mode, the Smartdop 45 senses the reflection of light from the hemoglobin of the red blood cells in subcutaneous vessels by utilizing infrared light and allows the observation of blood volume variations.^26^

**Figure 1:**
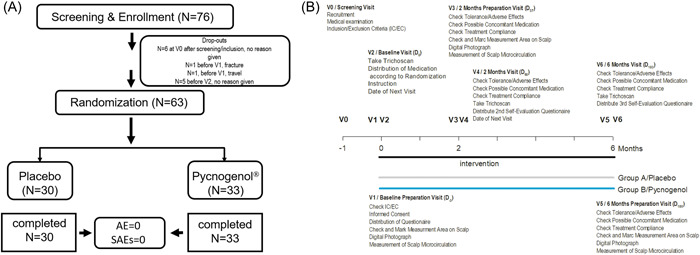
(A) An overall trial design showing number of subjects who finished the study and number of subjects who withdrew including reasons for withdrawal. (B) Details for both groups of the randomized, placebo‐controlled, double blinded study testing the efficacy of the oral supplement Pycnogenol® versus a placebo. At visit 0 (V0) demographic data were obtained, and a medical examination was done. At V1, informed consent was obtained, test area was marked, digital photograph was taken, microcirculation was measured, hair was colored in the test area. At V2, digital photographs were taken, subjects were allocated to either of the two groups A/placebo or B/Pycnogenol,® received medication and were assessed for skin physiological parameters as indicated.

After these baseline assessments, volunteers were randomly assigned to two groups: The placebo and the Pycnogenol® group (Figure 1A,B). After 2 months and 6 months, skin physiological measurements identical to the ones performed at baseline were repeated. At each visit patients were reminded not to change their food habits and to restrain from the intake of any other food supplement. The 76 subjects enrolled were distributed according to an online block randomization service which had been provided by the sponsor; block size was 4, allocation ratio 1:1, 63 females finished the study and 13 dropped out. The mean age of the placebo group (*N* = 30) and the Pycnogenol® group (*N* = 33) was 54 years. Subjects and investigators were unaware of the treatment. The selection of the actual study design is based on previous studies, where similar doses and treatment modalities resulted in significant effects on study outcomes during a time frame of 2 to 3 months.^23^, ^27^

### 2.4 Statistical analysis

Normal distribution of the data was tested using the Shapiro−Wilk test. For comparison of significant differences within each group, the one way analysis of variance (ANOVA) with a Student−Newman−Keuls post hoc test or the corresponding rank test, Kruskal−Wallis one way ANOVA on ranks (Dunn's) was used. Alternatively, for comparison between the groups, the change between reading point and baseline (D_−3_) was calculated. All statistical tests were considered statistically significant if the *p*‐value was less than 0.05. SigmaPlot 14.0 (Systat Software GmbH) was used for statistical analysis and graph design. The data are presented in box plots giving median (black line) and mean (red line), if relevant due to statistical test. The boundary of the box closest to zero indicates the 25th percentile, and the boundary of the box farthest from zero indicates the 75th percentile. Whiskers (error bars) above and below the box indicate the 95th and 5th percentiles. Circles reflect outliers.

## 3 RESULTS

In this randomized, placebo‐controlled, double blind intervention study, we assessed the effect of a nutritional supplement consisting of 70 ± 5% procyanidins built from condensed monomers of catechin and epicatechin in a dose 3 × 50 mg Pycnogenol® a day over a period of 6 months on hair loss in Chinese menopausal women. From the 76 women initially enrolled in the study, 13 subjects withdrew from the study before distribution of medication on visit 2. Thus, the final number of subjects which completed the study was 63 with a mean age of 54 years as shown in Figure 1A. The study design is depicted in Figure 1B. The drop‐out rate was similar as observed for other intervention studies employing Pycnogenol as add‐on in comparison to best treatment taking 6 months or longer.^28^, ^29^, ^30^, ^31^

**Figure 2:**
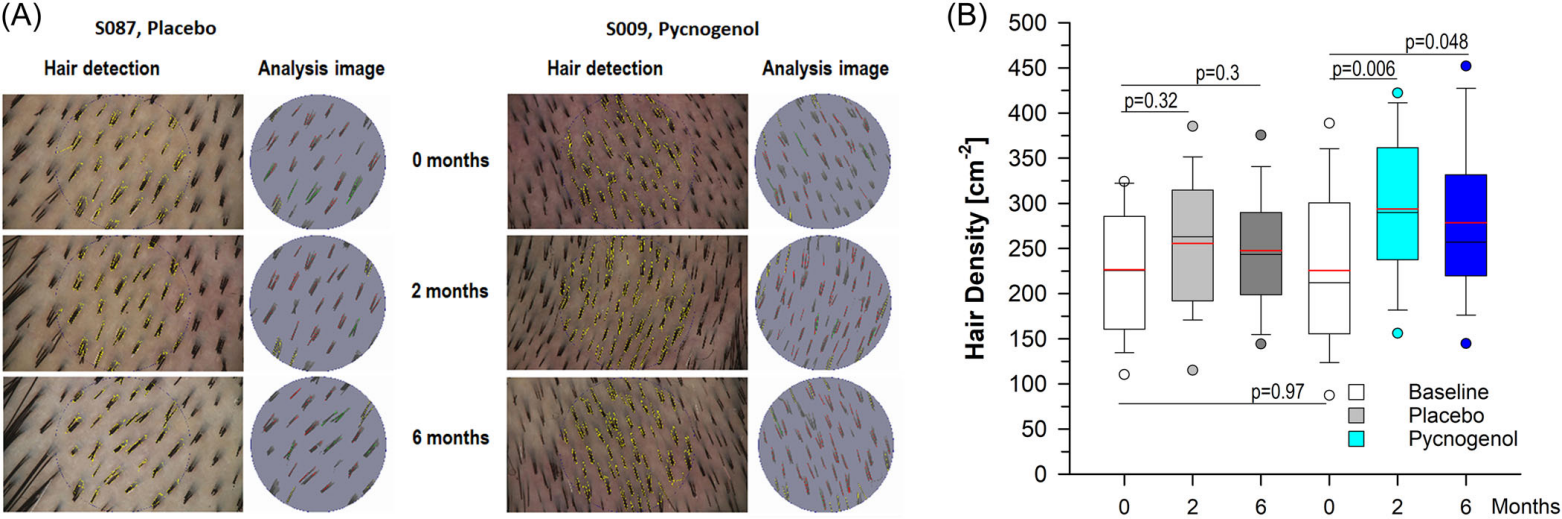
Results for hair density are shown (A) as epiluminescence microscopy (elm) photographs and the corresponding Trichoscan evaluations from representative examples of the placebo and the Pycnogenol® group at the beginning and after 2 and 6 months and (B) as box plots with median (black line) and mean (red line) for both groups obtaining either placebo (*N* = 30) or Pycnogenol® (*N* = 33) as indicated. The boundary of the box closest to zero indicates the 25^th^ percentile, and the boundary of the box farthest from zero indicates the 75^th^ percentile. Whiskers (error bars) above and below the box indicate the 95^th^ and 5^th^ percentiles. Circles reflect outliers. One way analysis of variance Student−Newman−Keuls (SNK, red line reflects mean), *p < 0.05 versus untreated.

Hair density, detected by the validated Trichoscan analysis from digital photographs, was assessed in both treatment groups at the beginning and after 2 and 6 months of treatment (Figure 2A).^32^ Mean (SD) hair density at the beginning was 226.6 (69.9) hairs/cm^2^ in the placebo group and 225.8 (87.0) hairs/cm^2^ in the Pycnogenol® group. Density increased in both groups over time by number. In the placebo group, a mean of 255.6 (76.1) after 2 months and of 247.7 (64.6) after 6 months was detected. In the Pycnogenol® group, the mean density increased to 293.6 (78.3) after 2 months and to 278.6 (87.6) after 6 months. One‐way ANOVA of all hair density data followed by pairwise multiple comparison (Student−Newman−Keuls) reflected nonsignificant increases by 13% (*p* = 0.321) and 9% (*p* = 0.295) in the placebo‐treated volunteers and significant increases in hair density of 30% (*p* = 0.006) and 23% (*p* = 0.048) in the Pycnogenol®‐taking women.

To detect potential changes in microcirculation, resting flux on the scalp was measured in both groups at the beginning and after 2 and 6 months of treatment by reflection photoplethysmography employing a PPG PG‐21 probe (Figure 2B). The latter measures volumetric changes in blood in peripheral microcirculation. The reflected light correlates to the changes in blood flow given as arbitrary units and has been used to confirm vascular alterations in alopecia areata.^33^ In the placebo‐treated volunteers, the median value at the beginning was 1.95 (1.48−3.50, 25% and 75% percentiles) and it slightly decreased to 1.85 (0.90−2.85) after 2 months and 1.55 (0.98−2.33) after 6 months. This corresponds to a decrease of flux of 5% and 20%, respectively, which was not significant as compared to the baseline. In the Pycnogenol® taking females, the median blood volume flux started with 2.30 (1.60−4.45) and it declined over time to 1.80 (1.35−2.80) and to 1.30 (0.90−2.50), which means a decrease of 21% and 44% after 2 and 6 months of treatment, respectively. Of note, the effect after 6 months was significantly smaller as compared to the baseline.

The effects of Pycnogenol® intake on scalp skin physiological parameters such as skin hydration and TEWL were also studied. Hydration of scalp skin was not significantly affected by Pycnogenol® or placebo intake. Given as median (25%−75% percentiles) the following results were obtained: In the placebo group, we measured a change of 1.5 (−6.50 to 11.25) after 2 months and a change of 8.5 (−8.25 to 23.0) after 6 months. In the Pycnogenol® group, no change of hydration (0, −15.0 to 6.0) was observed after 2 months and after 6 months there was an increase of 2 (−16.5 to 10.0).

**Figure 3:**
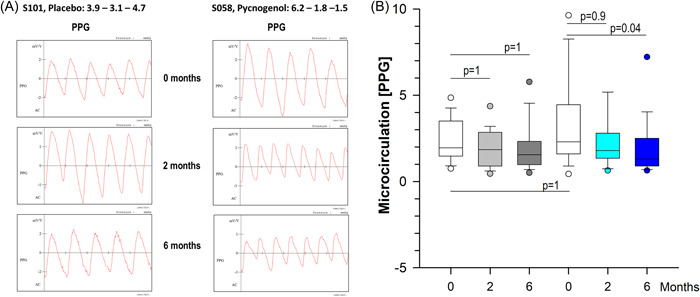
Results for microcirculation are given (A) as photoplethysmograms (PPG) from representative examples of the placebo and the Pycnogenol® group at the beginning and after 2 and 6 months and (B) as box plots with median (black line) for both groups obtaining either placebo (*N* = 30) or Pycnogenol® (*N* = 33) as indicated. The boundary of the box closest to zero indicates the 25^th^ percentile, and the boundary of the box farthest from zero indicates the 75^th^ percentile. Whiskers (error bars) above and below the box indicate the 95^th^ and 5^th^ percentiles. Circles reflect outliers. Kruskal−Wallis one way analysis of variance on ranks (Dunn's) was used. *p < 0.05 versus untreated.

In contrast, TEWL measurements revealed significant effects of Pycnogenol,® but not of placebo intake (Figure 3). Accordingly, a significant decrease of TEWL values of −2.5 (−4.4 to 0.1) was detected after 2 months Pycnogenol® intake, but not after placebo treatment, for which an increase of 6.0 (0.5−9.3) was determined. Kruskal−Wallis one‐way ANOVA on ranks indicated a significant difference between ΔTEWL from placebo and Pycnogeol after 2 months of intake (Dunn's method, *p* = 0.001). After 6 months of intervention, placebo treatment resulted in an increase of 4.8 (−0.6 to 10.3) and Pycnogenol® in an increase of 1.0 (−0.8 to 1.3), and these effects were not significant (*p* = 0.573).

**Figure 4:**
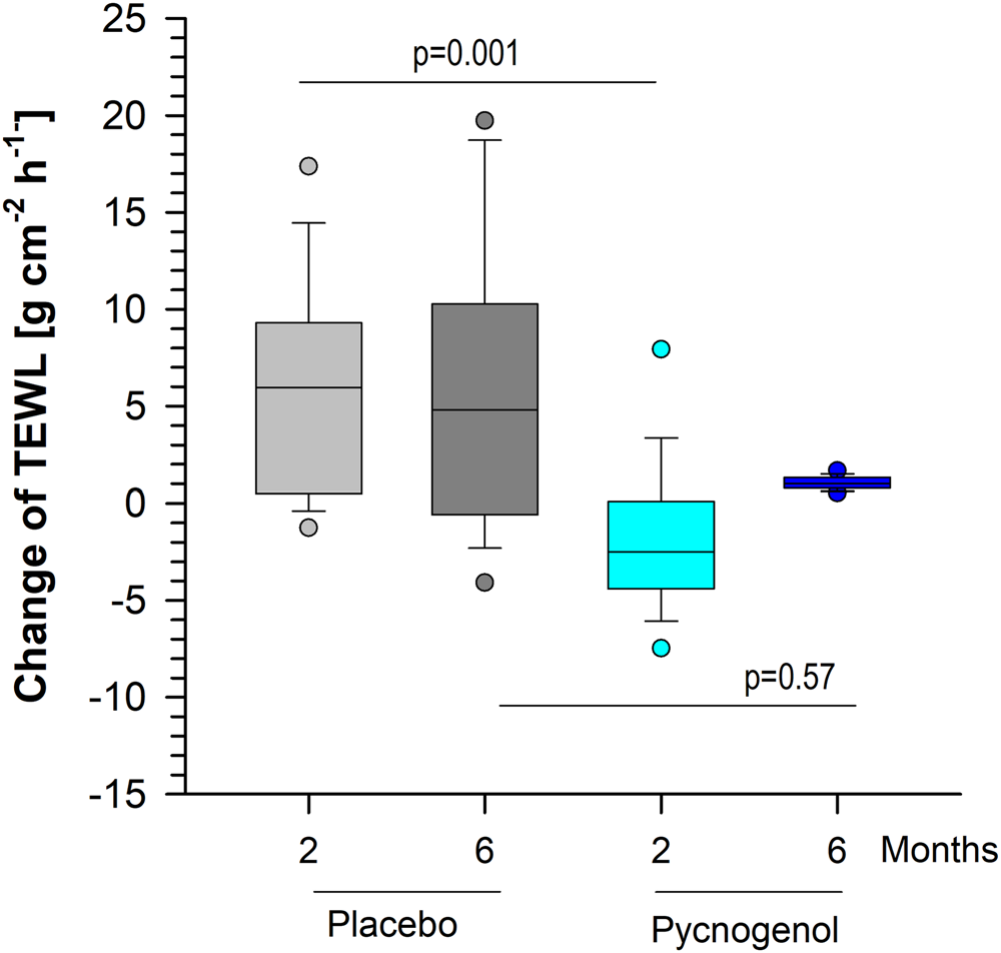
The change of transepidermal water loss (TEWL) is given as box plots with median (black line) for both groups obtaining either placebo (*N* = 30) or Pycnogenol® (*N* = 33) as indicated. The boundary of the box closest to zero indicates the 25^th^ percentile, and the boundary of the box farthest from zero indicates the 75^th^ percentile. Whiskers (error bars) above and below the box indicate the 95^th^ and 5^th^ percentiles. Circles reflect outliers. Kruskal−Wallis one way analysis of variance on ranks (Dunn's) was used. *p < 0.05.

Adverse events and serious adverse events were not observed during the whole study. Scalp conditions such as dandruff, greasy scalp, dry scalp, psoriasis, burning sensation, prurigo, inflamed scalp, eczema, and tautness were addressed in investigator's questionnaires at the beginning and after 6 months. None of the listed medical conditions were either present at the beginning of the study nor did they develop during the study (Figure 4).

## 4 DISCUSSION/CONCLUSION

We here report that daily intake of Pycnogenol® over 2 and 6 months significantly increased hair density in postmenopausal Han Chinese women, while the intake of placebo failed to do so. The increase of hair density by trend within the placebo group might be explained by the seasonality of hair growth, showing a maximal proportion in telogen hair in July.^13^ As the study started in the end of August, an increased loss of hairs might have affected the hair density at the time of study start, when it might have been lower than in other seasons.

The precise mechanism by which Pycnogenol® intake improves hair density is currently not known. Of note, we observed that the beneficial effects of Pycnogenol® on hair density were associated with a decrease in steady state flux of scalp skin. Previous reports indicate that such a decrease might reflect an improvement of microcirculation in the skin: Clinical efficacy of low dose (150 mg/day) and high dose (300 mg/day) Pycnogenol® intake for 8 weeks was studied compared to a diosmin and hesperidin containing nutritional supplement, Daflon,® in patients suffering from chronic venous insufficiency.^17^ All microcirculatory parameters such as skin flux in supine resting position and capillary filtration showed a significant decrease at the end of Pycnogenol® intake which was significantly larger as compared to Daflon® and which correlated with an improvement in the symptomatic venous score and an increase in pO_2_ as well as a decrease in pCO_2_.^17^ Also, the underlying effect of Pycnogenol® on endothelium‐dependent vasodilatation was addressed in a placebo‐controlled study enrolling 16 young, healthy Japanese men. Intake of 180 mg/day for 2 weeks did not affect clinical characteristics such as systolic and diastolic blood pressure and forearm blood flow in general. Effects on endothelial function were studied by modulation of forearm blood flow before and after intake of Pycnogenol® versus placebo by infusion of the endothelium‐dependent vasodilator acetylcholine, showing a significant increase of blood flow only under Pycnogenol® treatment but not after infusion of an endothelium‐independent vasodilator such as sodium nitroprusside.^34^ As addition of the NO synthase inhibitor N^G^‐monomethyl‐l‐arginine was able to completely inhibit the effect of Pycnogenol® on forearm blood flow in response to acetylcholine, it was suggested that Pycnogenol® might impact endothelium‐dependent vasodilatation by increasing NO production.^34^ Similarly, Pycnogenol intake significantly decreased the wheal and flare response to a local histamine injection in young females assessed as wheal area, redness and time frame until complete wheal disappearance, which was accompanied by a significant decrease in capillary permeability detected by straingauge plethysmography.^26^ In addition, flow mediated dilatation in patients with stable coronary artery disease was significantly increased after intake of 200 mg/day Pycnogenol® for 8 weeks, as was shown in a double‐blind, randomized, placebo‐controlled, cross‐over study.^23^

In addition, we observed a transient decrease of TEWL after 2 months in the Pycnogenol®‐taking females. Similarly, a significant improvement of TEWL was recently shown for urban outdoor workers in a randomized, placebo‐controlled, double blind, cross‐over intervention study after taking 2 × 50 mg Pycnogenol® over a period of 3 months in autumn in Shanghai.^35^ In that study, a significant effect of Pycnogenol® on TEWL was only observed in the group, consuming the Pycnogenol® in the moderate season pointing to the seasonality of that parameter.

Oxidative stress may also contribute to the pathophysiology of FPHL as shown for (i) dermal papilla cells from frontal as compared to occipital scalps of androgenetic alopecia patients in vitro^36^ and for (ii) scalp scrapes from affected patients and healthy controls presenting significant higher activity of superoxide dismutase, and catalase, or higher levels of reduced glutathione and malondialdehyde in vivo.^37^ Therefore, the antioxidative capacity of Pycnogenol® might also contribute to the observed positive effects on hair density.

The question arose whether Pycnogenol,® might be safe for users complaining about a disease with immunological background. In this regard it can be stated that the investigated nutritional supplement has been in use since 1970 in Europe with no reports of serious adverse events.^19^

In the aggregate, these reports and the present study corroborate the ameliorating effect of Pycnogenol® on microcirculation. Given the fact that daily Pycnogenol® intake was well tolerated, the present study extends the spectrum of potential indications for Pycnogenol® intake to FPHL.

## AUTHOR CONTRIBUTIONS

**Carr Cai**: investigation; project administration. **Bill Zeng**: investigation; project administration. **Lydia Lin**: investigation; project administration; validation. **Miranda Zheng**: investigation; project administration; supervision; validation. **Carolina Burki**: resources. **Susanne Grether‐Beck**: formal analysis; investigation; visualization; writing − original draft; writing − review & editing. **Jean Krutmann**: conceptualization; writing − original draft; writing − review & editing.

## CONFLICTS OF INTEREST

I. M., B. Z., C. C., and M. Z. are stuff scientists of Intertek Testing Services Ltd. C. B. is director of product development with Horphag. S. G. B. is a stuff scientist in the IUF and did not obtain any sponsoring. J. K. serves as a consultant to Horphag Research.

## ETHICS STATEMENT

The study was approved by the local ethical committee. All study subjects signed an informed consent form after the nature and the purpose of the study had been explained to them.

## TRANSPARENCY STATEMENT

The lead author Jean Krutmann affirms that this manuscript is an honest, accurate, and transparent account of the study being reported; that no important aspects of the study have been omitted; and that any discrepancies from the study as planned (and, if relevant, registered) have been explained.

## Data Availability

The data that support the findings of this study are available on request from the corresponding author. The data are not publicly available due to privacy or ethical restrictions.
